# Red cell distribution width as a predictor of multiple organ dysfunction syndrome in patients undergoing heart valve surgery

**DOI:** 10.1242/bio.036251

**Published:** 2018-08-20

**Authors:** Piotr Duchnowski, Tomasz Hryniewiecki, Mariusz Kuśmierczyk, Piotr Szymanski

**Affiliations:** 1 Institute of Cardiology, Department of Acquired Cardiac Defects, 04-628 Warsaw, Poland; 2 Institute of Cardiology, Department of Cardiosurgery and Transplantology, 04-628 Warsaw, Poland

**Keywords:** Valve surgery, Risk stratification, Red cell distribution width, Multiple organ dysfunction syndrome

## Abstract

The aim of the study was to evaluate the prognostic value of red cell distribution width (RDW) for multiple organ dysfunction syndrome (MODS) in the early postoperative period in patients undergoing valve replacement or repair surgery. A prospective study was conducted on a group of 713 patients with haemodynamically significant valvular heart disease who underwent elective valvular surgery. The primary end-point at the 30-day follow-up was postoperative MODS. The secondary end-point was death from all causes in patients with MODS. The postoperative MODS occurred in 72 patients. At multivariate analysis: RDW (OR 1.267; 95% CI 1.113-1.441; *P*=0.0003), creatinine (OR 1.007; 95% CI 1.001-1.013; *P*=0.02) and age (OR 1.047; 95% CI 1.019-1.077; *P*=0.001) remained independent predictors of the primary end-point. Receiver operator characteristics analysis determined a cut-off value of RDW for the prediction of the occurrence of the perioperative MODS at 14.3%. RDW (OR 1.448; 95% CI 1.057-1.984; *P*=0.02) and age (OR 1.057; 95% CI 1.007-1.117; *P*=0.04) were associated with an increased risk of death in patients with perioperative MODS. Elevated RDW is associated with a higher risk of MODS and death in patients with MODS following heart valve surgery.

## Abstract (precis)

**Summary:** This is a single-centre study with a limited number of patients. The results of our research may be helpful in the perioperative strategy in patients undergoing heart valve surgery.

## INTRODUCTION

Postoperative multiple organ dysfunction syndrome (MODS) is a complication that may occur after heart valve surgery, which significantly increases the risk of hospital death. The pathophysiologic basis for the postoperative MODS is cellular damage, which is manifested when cellular repair does not occur (Waxman, 1987). In the available literature, information on the risk factors of postoperative MODS in patients undergoing heart surgery is limited. Among these factors, preoperative NYHA functional class, prolonged mechanical ventilation, perioperative hypoxia, time of aortic cross-clamping, older age, surgery on cardiac arrest, severe left ventricular dysfunction and elevated value of creatinine are the most frequently described (Yuan et al., 2018; Litwiński et al., 2018; Fernandez-Zamora et al., 2018; Yeung et al., 2016; Zhao et al., 2016; Eremenko and Minbolatova, 2015; Belletti et al., 2017).

Red cell distribution width (RDW) is a measure of the variability of the size of red blood cells. RDW is calculated manually or automatically by dividing the standard deviation of red blood cell volume and the volume of red blood cells expressed as a percentage. Higher values of RDW are a result of ongoing inflammation, increased destruction of red blood cells or red blood cell production dysfunction related to a deficiency of iron, folic acid or vitamin B12 (Poludasu et al., 2009; Montagnana et al., 2011; Salvagno et al., 2015; Aslan et al., 2002). Previous studies have indicated elevated RDW as an applicable parameter in the risk assessment and determination of prognosis in patients with aortic stenosis and perioperative stroke following heart valve surgery (Duchnowski et al., 2016, Duchnowski et al., 2017). The usefulness of the RDW as a predictor of perioperative MODS in patients undergoing valve surgery is currently unknown. Therefore, we attempted to check the prognostic value of RDW in anticipation of the MODS in this group of patients.

## RESULTS

**Fig. 1.**
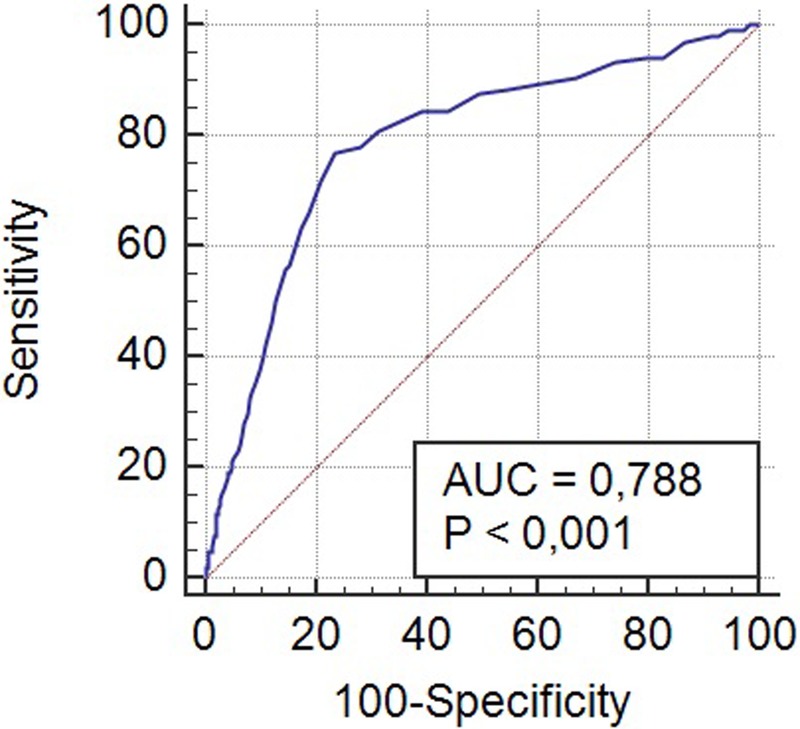
**Area under receiver operating characteristic (ROC) curve of RDW for a multiple organ dysfunction syndrome following valve replacement/repair surgery.**

The study included 713 patients who underwent heart valve surgery with or without concomitant procedures on coronary arteries. The mean age in the study group was 63.1 (±12.9). Fifty-three (7.4%) patients had significantly impaired left ventricular systolic function (ejection fraction ≤35%). The mean RDW level was 13.8 (standard deviation [s.d.]±1.6). Table 1 shows the preoperative characteristics of the patients studied. A multiple organ dysfunction syndrome occurred in 72 patients (44 patients required renal replacement therapy, 45 patients prolonged mechanical ventilation, 43 patients supply of catecholamines and 23 patients mechanical circulatory support; extracorporeal membrane oxygenation or an intra-aortic balloon pump). In eleven patients stroke occurred. Computed tomography scans in 10 patients with postoperative stroke showed multiple disseminated ‘hypodensity’ lesions in deep brain structures. The statistically significant predictors of perioperative MODS at univariate and multivariate analysis are presented in Table 2. RDW [odds ratio (OR) 1.267; 95% confidence interval (CI) 1.113-1.441; *P*=0.0003], creatinine (OR 1.007; 95% CI 1.001-1.013; *P*=0.02) and age (OR 1.047; 95% CI 1.019-1.077; *P*=0.001) remained independent predictors of the primary end-point. The optimal cut-off point for MODS was calculated at 14.3% RDW. The area under receiver operator characteristic curve for postoperative MODS for RDW is 0.788 (95% CI 0.715–0.828) (Fig. 1A). A positive correlation was found between the level of lactates measured immediately after surgery and preoperative RDW (r=0.38; *P*=0.01). In a further follow-up MODS in 38 patients led to death. Statistically significant predictors of death from all causes in patients with perioperative multiple organ dysfunction syndrome at univariate and multivariate analysis are presented in Table 3. At multivariate analysis, age (OR 1.057; 95% CI 1.007-1.117; *P*=0.04) and RDW (OR 1.448; 95% CI 1.057-1.984; *P*=0.02) remained predictors of mortality.

**Table 1.**
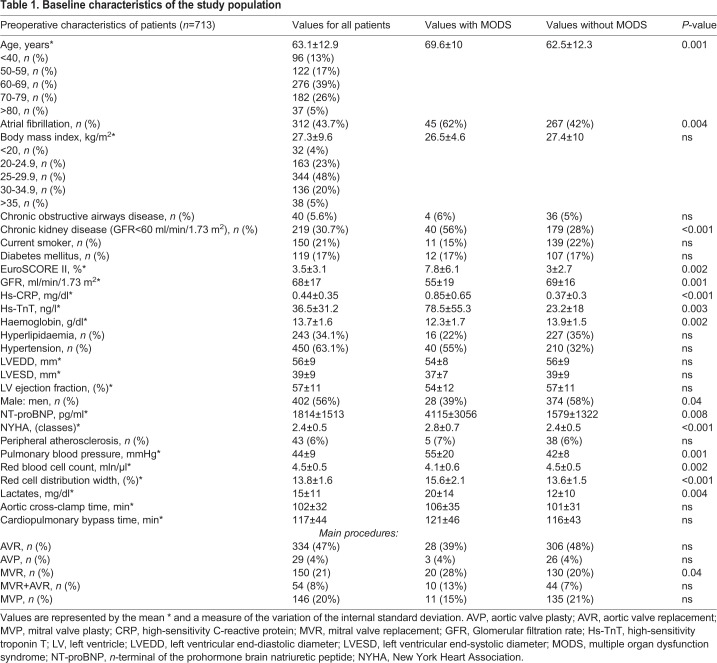
**Baseline characteristics of the study population**

**Table 2.**
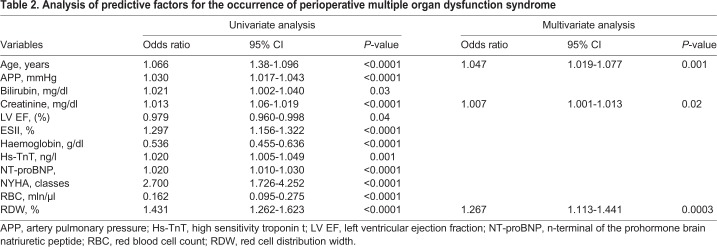
**Analysis of predictive factors for the occurrence of perioperative multiple organ dysfunction syndrome**

**Table 3.**
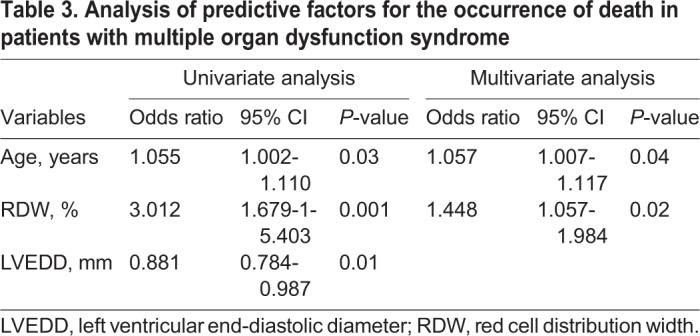
**Analysis of predictive factors for the occurrence of death in patients with multiple organ dysfunction syndrome**

## DISCUSSION

Heart valve surgery is often the only way to improve the functioning and life extension of patients with heart valve disease. Unfortunately, this treatment is associated with the risk of serious postoperative complications, including multiple organ dysfunction syndrome. The aim of this study was the identification and evaluation of selected biomarkers and comorbidities, and their ability to predict postoperative MODS in the early postoperative period in patients treated surgically because of valve disease.

In the present work, RDW remained an independent predictor of postoperative MODS and MODS-related mortality among 713 patients undergoing heart valve surgery. RDW is a widely accessible and a simple parameter designated for each patient during a standard blood test. So far, predictive ability of the RDW in various cardiovascular disorders has been reported in numerous publications (Dabbah et al., 2010; Kojima et al., 2015; Allen et al., 2010; Kim et al., 2012). However, in the available literature, information regarding the usefulness of RDW parameter in patients undergoing heart valve surgery is limited. Two reports, describing 191 and 500 patients undergoing heart valve surgery, demonstrated a significant correlation between elevated RDW and an increased risk of death and perioperative stroke (Duchnowski et al., 2016, 2017).

To the best of our knowledge, there are no reports describing the usefulness of the RDW in anticipation of the MODS in the early postoperative period. There have been reports that described a RDW as a predictor of death in patients with MODS*.* Oh et al. demonstrates that RDW is an independent predictor of mortality in patients with acute kidney injury treated with continuous renal replacement therapy (Oh et al., 2012). The available literature report that RDW is a predictor of mortality in patients with acute pancreatitis, septic shock and acute ischemic stroke, as well as a poor stem cell mobilization in patients with advanced chronic heart failure (Şenol et al., 2013; Kim et al., 2015; Ani and Ovbiagele, 2009; Poglajen et al., 2015). It also showed a significant correlation between scoring system such as Glasgow coma scale (GCS), global registry of acute coronary events risk score (GRACE) and the values of the RDW (Kara et al., 2015, Chang et al., 2018).

The mechanisms explaining the relationship between the increase in RDW values and worse prognosis are unexplained. Some authors suggest that large immature erythrocytes present in the circulatory system – which increase RDW – are the cause of impaired microcirculation. As they age, erythrocytes gradually lose the ability to deform the cell membrane. This feature is very important during the squeezing of erythrocytes through vessels of a small diameter. Too rigid and brittle erythrocytes observed in patients with elevated values RDW cannot squeeze through the capillaries and thus impair blood flow through the microcirculation and block small blood vessels, leading to organ ischemia (Patel et al., 2013). This hypothesis can be confirmed by the positive correlation between preoperative value of RDW and postoperative lactate concentration as well as hypodensity lesions in deep brain structures shown in computed tomography.

On the other hand, some authors suggest that RDW is an indicator of a patient's physiologic reserve – the ability of cells to defend against the strong stress of hypoxia (Hunziker et al., 2012; Bazick et al., 2011; Bion, 2000). Physiological reserve is very important in extremal situations, such as heart valve surgery. Elevated RDW, meant to reflect a reduced physiological reserve, may explain the fact of a higher incidence of postoperative MODS.

## MATERIALS AND METHODS

The current prospective study was performed on consecutive patients with hemodynamically significant valve defects (aortic stenosis, aortic regurgitation, mitral stenosis and mitral regurgitation) with no porcelain aorta who underwent elective replacement or repair of the valve at the Institute of Cardiology, Warsaw, Poland. The exclusion criteria were: a lack of consent to participate in the study, patients under 18 years of age, autoimmune diseases, chronic inflammatory bowel, active neoplastic diseases and active endocarditis. The risk of surgery using EuroSCORE II was calculated for each patient. The day before surgery a blood sample for biomarkers was collected from each patient. Complete blood count was performed with K2-EDTA samples, using a Cobas 6000 electronic counter (Roche, Mannheim, Germany). All procedures were performed through a midline sternotomy incision under general anaesthesia in a normothermia. The primary end-point was multiple organ dysfunction syndrome defined as the dysfunction of two or more organs – central nervous system, cardiovascular system, respiratory failure, liver failure or renal failure – based on clinical examination, imaging test, laboratory parameters and/or the need to use organ replacement therapy. The secondary end-point was death from all causes in patients with MODS. Patients were followed up on for 30 days or until death. The follow-up of patients was conducted through direct daily observation during hospitalization and clinic visits 30 days after surgery. The study was conducted at the Institute of Cardiology, Warsaw. The protocol was approved by The Institutional Ethics Committee, number 1504.

### Statistical analysis

A statistical analysis was performed using SAS version 9.2. Data are presented as the mean±s.d. and the frequency (%). Shapiro-Wilk's test of normality was used to test the sample distribution. Intergroup comparisons were made using the Mann–Whitney *U*-test, the Pearson's χ^2^ test or Student’s *t*-test. Logistic regression was used to assess relationships between variables. The following preoperative covariates: age, aortic cross-clamp time, atrial fibrillation, body mass index, cardiopulmonary bypass time, chronic kidney disease, chronic obstructive airway disease, coronary artery disease, creatinine, EuroSCORE II, high-sensitivity C-reactive protein (hs-CRP), high-sensitivity troponin T (hs-TnT), hematocrit, haemoglobin, hypertension, left ventricular end-diastolic diameter (LVEDD), left ventricular end-systolic diameter (LVESD), left ventricular ejection fraction (LVEF), mean corpuscular haemoglobin, mean corpuscular haemoglobin concentration, mean corpuscular volume, New York Heart Association (NYHA) classes, N-terminal pro-hormone of brain natriuretic peptide (NT-proBNP), peripheral atherosclerosis, platelets, pulmonary blood pressure, red blood cell count (RBC), red cell distribution width (RDW), stroke history, tricuspid annulus plane systolic excursion (TAPSE) and white blood cell count were investigated for association with the endpoints in univariate analysis. Significant determinants (*P*<0.05) identified from univariate analysis were subsequently entered into multivariate models. Predictive value of RDW was assessed by a comparison of the areas under the receiver operator characteristics of the respective curve. On the basis of the Youden index, a cut-off point was determined that met with the criterion of maximum sensitivity and specificity for postoperative MODS. For the analysis of perioperative MODS in all the patient groups, the Kaplan–Meier curves were used. The value cut-off point and the log-rank test to compare curves were employed.
